# Genetics of body fat mass and related traits in a pig population selected for leanness

**DOI:** 10.1038/s41598-017-08961-4

**Published:** 2017-08-22

**Authors:** Henry Reyer, Patrick F. Varley, Eduard Murani, Siriluck Ponsuksili, Klaus Wimmers

**Affiliations:** 1 Leibniz Institute for Farm Animal Biology, Institute for Genome Biology, Wilhelm-Stahl-Allee 2, 18196 Dummerstorf, Germany; 2 Hermitage Genetics, Sion Road, Kilkenny, Ireland

## Abstract

Obesity is characterized as the excessive accumulation of body fat and has a complex genetic foundation in humans including monogenic high-risk mutations and polygenic contributions. Domestic pigs represent a valuable model on an obesity-promoting high-caloric diet while constantly evaluated for body characteristics. As such, we investigated the genetics of obesity-related traits, comprising subcutaneous fat thickness, lean mass percentage, and growth rate, in a pig population. We conducted genome-wide association analyses using an integrative approach of single-marker regression models and multi-marker Bayesian analyses. Thus, we identified 30 genomic regions distributed over 14 different chromosomes contributing to the variation in obesity-related traits. In these regions, we validated the association of four candidate genes that are functionally connected to the regulation of appetite, processes of adipogenesis, and extracellular matrix formation. Our findings revealed fundamental genetic factors which deserves closer attention regarding their roles in the etiology of obesity.

## Introduction

Basic process involved in the development and metabolism of white adipose tissues are of high scientific importance, especially due to the impact of pathological excess of body fat mass on human health. Obesity and obesity related diseases, such as diabetes, high blood pressure and heart diseases, reach epidemic proportions worldwide with gravely consequences especially in industrialized and developing countries^1^.

Several hypotheses are widely discussed giving different explanations for the genetic manifestation of obesity in humans (reviewed by^2^). In agreement, obesity is a highly polygenic trait and heritability estimates of obesity related phenotypes (e.g. body mass index; BMI) in humans revealed a proportion of 30–70% assigned to genetics^3, 4^. Nevertheless, beside rare forms of obesity caused by monogenic high-risk mutations, the complex disease pattern is based on polygenic causes and suggested to be influenced by environmental factors, lifestyle, and dietary habits. Specifically, main driving factors are the permanent availability and consumption of energy dense foods combined with low levels of physical activity which potentially cause chronic imbalance between energy intake and expenditure^5^. Fundamental metabolic processes and accompanied genetic principles involved in the etiology of obesity related diseases are analogous in all mammalian species. Accordingly, commercial pigs represent an intact biological system on an obesity promoting high caloric diet throughout their lifetime coupled with low physical activity in the conventional production processes. Under these preconditions, pigs are highly susceptible for a disturbed fat metabolism and an obesity related phenotype but at the same time adapted to a diabetogenic environment^6^. However, due to the breeding structure and the continuous phenotypic evaluation of animals in terms of fat, muscle, and growth traits, basic molecular variations as well as minimal alterations in fat metabolism can be related to genetic causes. Therefore, the elucidation of the genetic architecture of traits related muscle and fat mass in these commercial pig lines provide interesting insights into major and minor factors contributing to the development of obesity related phenotypes.

Genetic studies in different mammalian species, e.g. humans, mice, and pigs, revealed a consistently large number of genes affecting fat deposition and the occurrence and dimension of obesity^7–9^. Specifically, major players like melanocortin 4 receptor (*MC4R*), leptin (*LEP*), and fat mass and obesity-associated gene (*FTO*) are known key regulators of feed intake/appetite and energy homeostasis^9^. As many of these factors were derived and validated by genome-wide analyses across different species, the present study aimed to identify genetic markers and genomic regions associated with fat, leanness, and growth traits in pigs. Exploiting the genetic resource of mammalian livestock species can provide positional and functional candidate genes beyond the general knowledge about the main sources for the genetic predisposition of obesity.

## Results

### Genome-wide association analysis of lean mass percentage (LMP)

**Figure 1:**
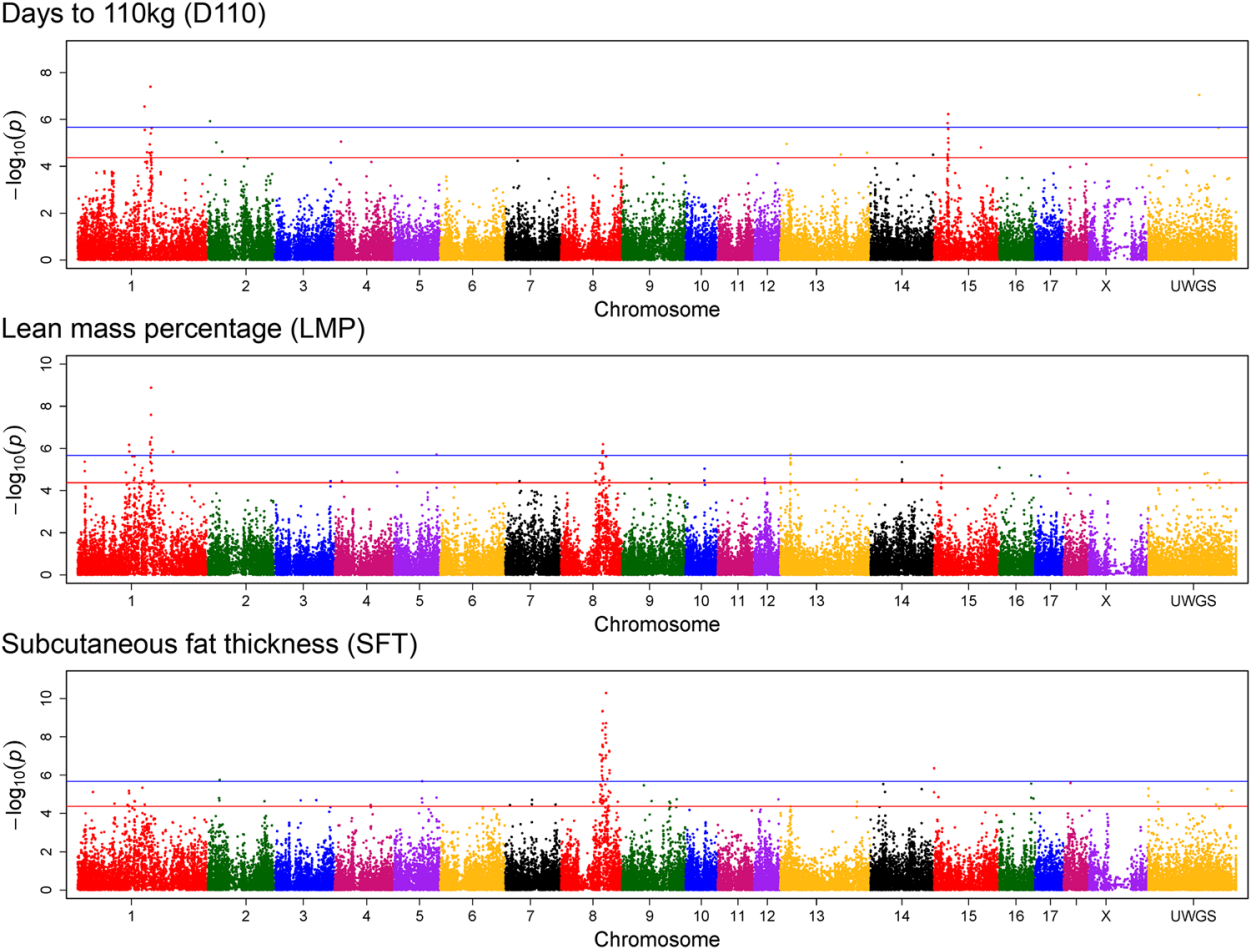
Manhattan plots of genome-wide association analysis results for traits related to body fat mass using a single marker (mixed linear model) approach. Chromosome ‘UWGS’ represents a contig of unmapped markers. The threshold for suggestive and genome-wide significance was set to *p* = 4.3e-05 (equals to -log_10_(p-value) = 4.37) and *p* = 2.1e-06 (equals to -log_10_(p-value) = 5.67), respectively.

**Figure 2:**
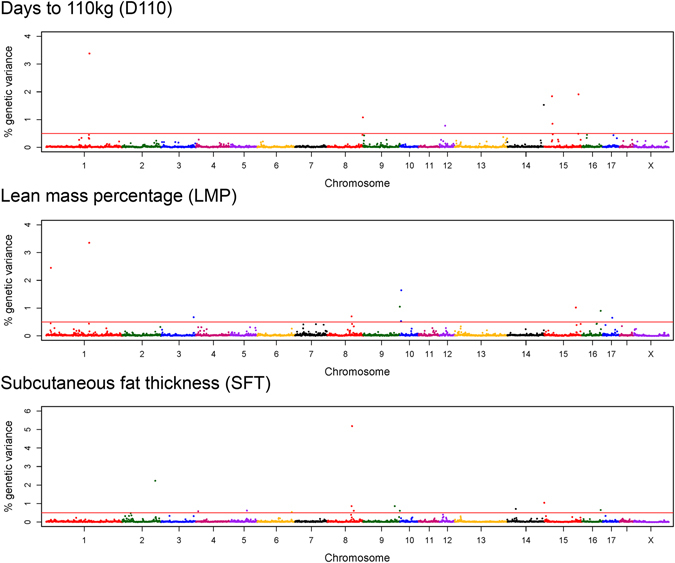
Manhattan plots depicting the proportion of 1 Mb genomic windows to the genetic variance of analysed traits obtained from a Bayesian multi-marker approach (Bayes B). The threshold line represents contributions to the additive genetic variance of traits above 0.5%.

The genome-wide analyses revealed 10 genomic windows with prominent contribution to the genetic variance in LMP (Table 1). In total, genetics explained 41% of the total variance in LMP. The 1-Mb (mega base pairs) window from 178.0 to 178.9 Mb on chromosome 1 explained 0.81% of the genetic variance of the trait. Supportively, single-marker analysis pointed to a genomic region between 175.6 and 180.5 Mb on chromosome 1 including 15 significantly associated markers, of which 7 single-nucleotide polymorphisms (SNP) exceed the threshold of genome-wide significance (*p* < 2.1e-06) (Fig. 1, Supplementary Table 2). The highest significantly associated SNP ALGA0006623 mapped next to the *MC4R* locus. Furthermore, a second region on chromosome 1 (19.0–20.0 Mb) explained the highest proportion of the genetic variance of LMP with 1.39% (Fig. 2). Exclusively, the uncharacterized ENSSSCG00000028974, with predicted sulfotransferase activity, is located in this 1-Mb window. A third genomic region on chromosome 1, at approximately 125 Mb, was indicated by 3 markers reaching genome-wide significance in single-marker analysis (Fig. 1). This region harbours several genomic features and significantly associated markers are linked to *MYO1E* (ALGA0005584), *ADAM10* (ALGA0005610) and novel protein coding features (MARC0103791). Another region linked to the variation in LMP was identified between 98.6 and 104.0 Mb on chromosome 8 by single-marker analyses (Fig. 1) and was further supported by a 1-Mb window between 100.2 and 100.9 Mb (Table 1). While the whole 5 Mb spanning region harbours in total 16 genetic features, none of the genes represented a clear positional or functional candidate for LMP. Single-marker but not multi-marker analyses revealed a region on chromosome 13 from 24.8 to 25.5 Mb harboring several functional candidate genes e.g. *ACAA*, *OXSR1*, and *SLC22A14* (Fig. 1). The integration of both genome-wide analysis approaches further revealed a genomic window between 77.0 and 77.9 Mb on chromosome 16 contributing to the genetic variance in LMP (0.55%). The most prominent SNP ALGA0091730 is located at 77.6 Mb next to *FAT2*, *SLC36A1* and ENSSSCG00000017082 (predicted as *SPARC*).

**Table 1:** Overview of genomic regions identified by integrating genome-wide single-marker (linear regression) and window-based (Bayes B) approaches for days to 110 kg (D110), lean mass percentage (LMP), and subcutaneous fat thickness (SFT).

Trait	Chr.	Genomic window				Top SNP in single-marker analysis				
		Start (Mb)	End (Mb)	Explained genetic variance (%)	Number of significant single marker	SNP	Position	p-value	Number of features in window	plausible candidate gene
D110	1	146.0	147.0	0.74	0	—	—	—	18	*GPR176*
	1	176.0	176.9	0.59	2	ASGA0004976	176492950	1.6e-07	6	*TNFRSF11A*
	1	177.0	178.0	0.62	2	H3GA0003111	177074934	1.2e-05	3	—
	1	179.0	180.0	1.8	1	MARC0013872	179327620	1.1e-05	13	*MC4R*
	8	145.0	146.0	0.53	0	—	—	—	7	—
	8	146.0	147.0	0.51	0	—	—	—	10	*BMP3*
	8	147.0	148.0	0.98	1	ASGA0040607	147636271	2.9e-05	5	*GK2*, *BMP2K*
	12	23.0	24.0	0.65	0	—	—	—	28	—
	14	151.0	152.0	1.5	1	M1GA0019945	151444665	3.7e-05	3	-
	15	32.0	32.9	1.38	2	ALGA0084616	32933373	3.8e-06	0	—
	15	142.0	143.0	1.57	0	—	—	—	11	*RHBDD1*
LMP	1	19.0	20.0	1.39	0	—	—	—	2	ENSSSCG00000028974 (*UST*)
	1	178.0	178.9	0.81	1	ALGA0006623	178024855	1.1e-09	2	*MC4R* at 178.5 Mb
	3	134.0	134.8	0.57	1	ALGA0021640	134815777	3.5e-05	9	*ATP6V1C2*
	7	118.0	119.0	0.51	0	—	—	—	7	*CALM1*
	8	100.2	100.9	0.52	6	ASGA0039385	100921095	1.9e-06	0	—
	9	152.1	153.0	0.69	0	—	—	—	0	—
	10	4.0	4.9	0.56	0	—	—	—	1	—
	10	5.0	6.0	0.98	0	—	—	—	1	*BRINP3*
	15	131.0	132.0	0.84	0	—	—	—	8	*PECR*
	16	77.0	77.9	0.55	1	ALGA0091730	77611594	1.5e-05	8	ENSSSCG00000017082 (*SPARC*)
SFT	2	137.0	138.0	2.12	1	ALGA0016010	137184334	2.6e-05	9	*SLC27A6*
	4	9.0	9.9	0.5	0	—	—	—	5	—
	5	68.0	69.0	0.6	2	MARC0036560	68326348	1.6e-06	10	*CCND2*
	6	143.0	144.0	0.6	0	—	—	—	6	*PRKAA2*
	8	100.2	100.9	0.7	9	CASI0009346	100889708	6.6e-10	0	—
	8	102.0	102.9	5.36	7	ALGA0048723	102070107	2.4e-09	3	—
	8	109.1	109.8	0.5	6	ALGA0122904	109553065	6.8e-11	8	*TRPC3*, *BBS7*
	9	131.1	132.0	1.0	1	ALGA0054936	131303382	3.0e-05	4	—
	9	152.1	153.0	0.5	0	—	—	—	0	—
	14	35.0	36.0	0.7	1	DRGA0013774	35264755	8.3e-06	6	ENSSSCG00000009839 (*CIT*)
	15	0.0	1.0	1.03	2	ALGA0083738	259597	5.9e-07	3	*TNFAIP6*
	16	77.0	77.9	0.6	2	ALGA0091730	77611594	2.5e-06	8	ENSSSCG00000017082 (*SPARC*)

### Genomic regions associated with subcutaneous fat thickness (SFT)

The estimated proportion of genetics to the total variance of SFT was 39.4%. In total, 12 1-Mb windows with contributions to the genetic variance above 0.5% were identified (Table 1). Three of these regions, on chromosome 8, 9, and 16, completely or partially overlap with regions linked to LMP. For the region on chromosome 8, results from single-marker analysis showed 59 significantly associated SNPs mapping in a 25 Mb spanning region between 94.4 and 119.8 Mb, of which 35 SNPs reached genome-wide significance (Fig. 1). The genomic window between 102.0 and 102.9 Mb explained the highest proportion of the genetic variance of SFT with 5.36% (Fig. 2). Based on the highest significantly associated SNP ALGA0122904, *TRPC3* and *BBS7* located at approximately 109.4 Mb were proposed as positional and functional candidate genes.

The 1-Mb genomic window on chromosome 16 showed a contribution of 0.6% to the genetic variance in SFT. The highest significantly associated marker obtained from single-marker analysis in this region mapped next to ENSSSCG00000017082 which is predicted as *SPARC* precursor. The second highest contribution to the genetic variance (2.12%; Fig. 2) in SFT was explained by a region between 137.0 and 138.0 Mb on chromosome 2. The most prominent single marker ALGA0016010 in this region mapped in an intergenic region between *SLC27A6* and *ISOC1*.

Furthermore, the results from single-marker analysis provide clues for at least two additional associated regions located at the proximal end of chromosomes 2 and 15 (Fig. 1). The region between 0.0 and 1.0 Mb on chromosome 15 was further supported by a 1-Mb window and pointed to *TNFAIP6* as positional candidate feature. The region on chromosome 2 was indicated by 3 significantly associated SNPs spanning the genomic region from 27.1 to 28.9 Mb. Specifically, the most prominent marker MARC0063133 mapped in an intronic region of the catalase encoding gene *CAT*.

### Genomic regions contributing to the variation in growth rate (D110)

For D110, genetic factors explained 42.2% of the total variance. Eleven 1-Mb genomic windows on 5 different chromosomes were identified (Table 1). Thereby, the highest proportion to the genetic variance of the trait was explained by a region between 179.0 and 180.0 Mb on chromosome 1 (Table 1). Additionally, 8 SNPs mapping between 174.9 and 179.4 Mb on chromosome 1 showed evidence for significant association with growth rate (Fig. 1, Supplementary Table 2). The highest significantly associated SNP ASGA0004992 mapped in an intergenic region between *CDH20* and *MC4R*. Thus, the associated region largely overlaps with results for LMP. The integration of both GWAS approaches further revealed a genomic region located on chromosome 15 as associated with D110 (Table 1, Fig. 1). In total, 6 SNPs showed significant associations with growth rate in the region between 32.4 and 34.7 Mb. The DNA segment around 34.3 Mb, in which most significantly associated SNPs are located, harbors two annotated genes, *TSN* and *NIFK*. A third region linked to D110 mapped between 147.0 and 148.0 Mb on chromosome 8. The window contributes 0.98% to the genetic variance of D110 and the highest significantly associated SNP ASGA0040607 is located between *GK2* and *PAQR3*. This region was further supported by two adjacent 1-Mb windows (145.0–147.0 Mb), each contributing above 0.5% to the genetic variance.

### Associations of selected candidate genes with obesity-related traits

Association analyses of SNPs located in *SLC27A6*, *SPARC*, *BBS7*, and *MC4R* were carried out in a representative subset of the analysed population (Table 2). For *SLC27A6*, *SPARC*, and *BBS7* significant associations with fat thickness and lean mass but not with growth rates were demonstrated. Highest significant associations were found between SFT and tagging SNPs of *SPARC* and *BBS7*, respectively. For both genes, the homozygous carriers of the rare allele (TT for *SPARC* and GG for *BBS7*) showed significant (*p* < 0.05) higher SFT. Specifically, compared to carriers of the major allele these individuals showed 13% and 9% higher levels of subcutaneous fat for *SPARC* and *BBS7*, respectively. Moreover, the observed effects of these two tagging SNPs on LMP were in the opposite direction with a similar order of magnitude. Association analyses of the genotyped *MC4R* polymorphism revealed significant associations with LMP but not with SFT. Moreover, the tagging SNP in *MC4R* showed a tendency for affecting growth rates, with carriers of the minor G-allele growing slower but having higher LMP.

**Table 2:** Association of selected candidate gene polymorphisms with days to 110 kg (D110), lean mass percentage (LMP), and subcutaneous fat thickness (SFT).

SNP	Trait	P-value	Least square means ± standard error (n)^1^						MAF^2^
*SLC27A6*			CC		CT		TT		
	SFT	**0.0486**	8.47^a^ ± 0.21	(42)	8.31^a^ ± 0.10	(187)	7.97^b^ ± 0.12	(127)	0.38
	LMP	**0.0323**	61.85^ab^ ± 0.28	(42)	61.83^a^ ± 0.18	(187)	62.32^b^ ± 0.21	(127)	
	D110	0.8097	138.02 ± 1.15	(42)	137.36 ± 0.89	(187)	137.43 ± 0.96	(127)	
*SPARC*			CC		CT		TT		
	SFT	**4.8E-05**	7.95^a^ ± 0.09	(214)	8.70^b^ ± 0.16	(74)	8.98^b^ ± 0.38	(13)	0.16
	LMP	**0.0005**	62.23^a^ ± 0.17	(214)	61.43^b^ ± 0.23	(74)	61.23^b^ ± 0.47	(13)	
	D110	0.2858	136.69 ± 0.64	(214)	135.46 ± 0.85	(74)	136.38 ± 1.65	(13)	
*MC4R*			GG		GA		AA		
	SFT	0.3601	8.00 ± 0.20	(51)	8.17 ± 0.11	(179)	8.33 ± 0.13	(124)	0.40
	LMP	**0.0011**	62.71^a^ ± 0.25	(51)	62.08^b^ ± 0.15	(179)	61.68^c^ ± 0.17	(124)	
	D110	0.0521	138.23^a^ ± 0.87	(51)	136.81^ab^ ± 0.55	(179)	135.97^b^ ± 0.63	(124)	
*BBS7*			GG		GA		AA		
	SFT	**2.1E-05**	8.55 ± 0.22	(42)	8.55 ± 0.13	(152)	7.82 ± 0.13	(145)	0.35
	LMP	**0.0039**	61.73 ± 0.29	(42)	61.73 ± 0.20	(152)	62.36 ± 0.21	(145)	
	D110	0.5066	138.16 ± 1.15	(42)	137.18 ± 0.89	(152)	136.97 ± 0.90	(145)	

^1^Least square means for genotypes were compared by *t*-test and *P*-values were adjusted by Tukey-Kramer correction. Superscripts ^a,b,c^ indicate for significant differences at P < 0.05. Significant associations (P < 0.05) are highlighted in bold. ^2^Minor allele frequency.

## Discussion

The current study elucidated the genetics of fat and muscle deposition in a pig population with a long-term breeding history of selection for leanness, growth and feed efficiency. Thus, it aims at the cross-species identification of genetic factors and genomic regions contributing to the genetic predisposition for an excess of body fat mass. Bayesian multi-marker approaches, which were shown to be beneficial to identify trait-associated genomic regions^10^, were applied to the dataset. Results were integrated with single-marker analyses which further provide supportive evidence for the association of complex traits and allelic variants^11, 12^. In total, the genome-wide scan for DNA segments influencing fat, muscle, and growth traits revealed 30 unique 1-Mb genomic windows located on 14 different chromosomes. Proposed candidate genes are involved in the mediation of processes related to extracellular matrix formation, fatty acid transport, carbohydrate metabolism, homeostasis, bone metabolism, and melanocortin system. As such, the analyses provide a major resource of prominent genomic regions and derived genetic factors with specialized functions in the context of fat metabolism and, moreover, with an array of complex contributions to the etiology of obesity-related diseases.

The results partially overlap with genetic factors previously reported in human and mouse studies dealing with the genetics of obesity (reviewed by^7^). In fact, the *MC4R* locus represents one of the major obesity-associated loci in humans^13, 14^ and is linked to fatness, growth, and efficiency traits in pigs^15^. However, the genotyping of the *MC4R* rs81219178 polymorphism in the current dataset and subsequent association analyses did not fully represent the highly significant associations obtained from the genome-wide analyses with LMP and D110. Other studies argue for at least a second genetic factor in this genomic region that influences fat deposition and/or muscle development as supported by conditional analysis including *MC4R* genotype information in pigs^16^. Moreover, for the human homologous gene, which is located on human chromosome 18, more than 130 mutations are described in the context of monogenic and polygenic obesity^17^. Depending on the localisation of the *MC4R* mutation, both response level and direction of effects on BMI vary, thus, arguing for a complex genomic structure of the *MC4R* locus with protective^18^ or causative^19^ implications in the context of obesity. Interestingly, in the current study, no significant associations between the *MC4R* rs81219178 polymorphism and STF were found. In agreement, *MC4R* genotypes observed in a long-term study in humans were shown to predominantly affect the level of weight traits, due to the regulation of appetite and food consumption, with more or less strong side and secondary effects on fat deposition^20^. However, although the *MC4R* locus has been intensively investigated in human and pigs for decades, the obtained results and the ambiguous situation in humans still argue for the careful dissection of the effects in this wide quantitative trait region. This is supported by several genome-wide analyses in humans consistently assigning variations in the *MC4R* locus to obesity-related traits^21, 22^ but having difficulties to clearly narrow down the molecular causes even in large meta-analyses.

The region on chromosome 8 that showed the highest contribution to the genetic variance of SFT harbours the *BBS7* gene, which is involved in the occurrence of the Bardet-Biedl Syndrome (BBS). Bardet-Biedl Syndrome is a developmental disorder which is independently caused by different genes and is characterised by diverse features including obesity^23, 24^. Specifically, *BBS7* plays a critical role in the assembly of BBS proteins to the BBSome complex. Beside obesity, molecular alterations of *BBS7* are shown to be associated with secondary clinical features like developmental delay and hypertension^25^. Moreover, associations of *BBS7* mutations and obesity were consistently observed in different population groups, as exemplified by analyses of a Russian^26^ and a Korean cohort^27^. Knock-out studies of genes that are involved in the pathophysiology of BBS, for instance *BBS7*, were shown to promote the obese phenotype due to altered feed intake and weight gain^28, 29^. Furthermore, recent findings argue for an impaired trafficking of the leptin receptor due to alterations in the BBSome^30^. With regards to the significant associations between the *BBS7* locus and fat thickness and leanness in pigs, this locus provides an interesting candidate not only for monogenic causes of obesity but also for its contribution to the polygenic implications on obesity rate.

The examined *SLC27A6* locus, also known as *FTP6*, coded for a fatty acid transporter which predominantly acts on the transport of palmitate and linoleate in the plasma membrane of heart cells^31^. Nevertheless, *SLC27A6* is also expressed in skeletal muscle^32^ and adipose tissue^33^. Evidence for an association of this locus with weight gain was previously obtained by genome-wide analyses in pigs^34^. Genetic variations in the porcine *SLC26A6* gene are related to divergent lipid metabolism processes^35^. Although little is known about the role of *SLC26A6* in the context of obesity, members of the fatty acid transport proteins are widely discussed as regulators of energy homeostasis, exogenous fatty acid uptake, and thermogenesis^36^.

The combined analyses further provided first evidence for an association of genetic variants of *SPARC* (ENSSSCG00000017082) with fat deposition. *SPARC*, also known as osteonectin, is highly conserved across species and the human homologous gene mapped on chromosome 5 at 151.6 Mb. In white adipose tissues, *SPARC* is known to be mainly located in the extracellular matrix (ECM). As such, *SPARC* is involved in the regulation of metabolic processes during adipogenesis and participates in the stabilisation of ECM structures through regulating the expression and modification of collagen^37, 38^. Beside the knowledge gap regarding genetic-based alterations of *SPARC*, the gene is well characterised in the background of obesity. Analyses in mouse models revealed a role of different *SPARC* isoforms in the systemic mobilization and migration of adipose stem cells and established interactions between *SPARC* expression and obesity related phenotypes^39, 40^. Furthermore, based on correlations between osteonectin plasma concentrations, *SPARC* expression and BMI, these connections were also validated in humans^41, 42^. On the functional level, osteonectin inhibits the differentiation of mesenchymal stem cells and preadipocytes into adipocytes and induce the production of osteoblasts by stimulating osteoblastogenesis^37^. Accordingly, *SPARC*-null mice showed no difference in weight traits but excessive accumulation of white adipose tissue due to increased size and number of adipose cells^43^. Phenotypic observations further revealed impaired infarct healing and collagen formation after myocardial infarction in *SPARC*-null mice indicating multi-dimensional functions of *SPARC* in adipose tissue organisation and cardio-vascular system^44^. Moreover, osteonectin represents a substantial proportion of non-collagenous proteins in mineralized tissues and is involved in bone calcification, collagen I deposition, fibrillogenesis, and bone turnover^45^. Interestingly, total lean mass was previously mentioned as predictor for bone mineral density^46^ and, thus, the current associations between *SPARC* and LMP provide evidence for a genetic link between *SPARC* mutations and altered bone metabolism. Consequently, in the context of recently discussed connections between osteoporosis and obesity^47, 48^, *SPARC* could act as an important mediator in the balance of adipogenesis and osteogenesis^49^.

The presented list of candidate genes and their functional contribution to the etiology of obesity highlighted the usability of genome-wide analysis as a valuable tool to elucidate the genetic architecture of complex traits across species. Nevertheless, the power of the approach is influenced by many factors such as population structure, confounding variables, and the informative value of analysed traits. With respect to these factors, animal models provide several advantages^50^.

Although the analysed pigs were not classified as having an obese phenotype, LMP and SFT varied considerably within the population enabling to identify genetic drivers for differences of body fat mass. Moreover, the analysed traits are more suitable compared to indirect measures like BMI to distinguish between genetic factors of fat or lean mass development^51^. Accordingly, the performed association analyses assigned alterations of leanness to the *MC4R* locus while less pronounced effects were observed for the association with fat thickness. Another advantage of translational studies in model organisms is the environmental stability within the population including homogeneous feeding regimes and the absence of confounding factors such as smoking and alcohol consumption. Moreover, the polygenic inheritance of obesity is suggested to be less complex in pigs due to the on-going selection based on body characteristics and subsequent fixation of loci^8, 51^. Consequently, there is less genetic heterogeneity which provides the possibility to uncover particular pathways and genetic factors contributing to the predisposition of obesity. The conducted genome-wide analyses of obesity-related traits in pigs revealed prominent genetic factors like *MC4R* and *BBS7* with known contributions to monogenic and polygenic causes in the etiology of obesity. The analyses provided a list of so far unknown and/or not examined candidate genes spotlighting pathways related to bone metabolism, extracellular matrix formation, and fatty acid transport. Genome-wide studies in livestock species provide supporting evidence for candidates discussed in humans and mouse and shed light into putative genetic factors with contribution to the polygenic cause of obesity. In particular, *SPARC*, as an interesting candidate gene involved in the bone-adipose axis, deserves further investigation with particular focus on human gene polymorphisms affecting obesity rate.

## Materials and Methods

### Data and phenotyping

All procedures described in this experiment were conducted under experimental licence from the Irish Department of Health in accordance with the Cruelty to Animals Act 1876 and the 1994 European Communities Regulations (Amendments of the Cruelty to Animals Act 1876). Data of boars of the Maxgro sire line recorded between 2006 and 2012 were provided by Hermitage Genetics (Kilkenny, Ireland). Animals have been grouped in standard commercial fully slatted pens which were mechanically ventilated to provide an ambient temperature of 18 °C with *ad libitum* access to feed and water. Diets had a net energy of 9.90 MJ/kg with 16.5% protein, 3.2% oil, 3.6% fibre and 4.8% ash as previously described^52^. Prior to the end of the feeding trial (at approximately 110 kg), subcutaneous fat thickness (SFT) and percent lean, as indicator for lean mass percentage (LMP), were measured between the 3rd and 4th last rib, 5 cm and 7 cm from the midline using a Piglog 105 ultrasonic device (Carometec A/S, Denmark). Individual growth rates were expressed as the number of days necessary to gain a final body weight of 110 kg (D110) and recoded for each animal. Descriptive statistics of the traits are presented in Table 3. Blood samples were taken from the *Vena jugularis* in EDTA containing tubes.

**Table 3:** Descriptive statistics of analysed obesity-related traits in a commercial pig population.

Traits (unit)	Abbreviation	N	Mean	SD	Minimum	Maximum
Days to 110 kg (d)	D110	861	138.99	5.93	124	167
Lean mass percentage (%)	LMP	861	62.08	1.55	56.3	66.3
Subcutaneous fat thickness (mm)	SFT	860	8.20	1.19	4.7	13.9

### Genotyping

Blood samples were used to extract DNA employing the QIAamp DNA Blood Mini Kit (Qiagen, Hilden, Germany). In total, 951 individuals were genotyped using porcine SNP60 Beadchips (Illumina, San Diego, CA, USA). Eleven samples were excluded from analyses due to sample call rates <0.97. In total, 861 individuals with complete phenotypic data were used. After filtering, 52920 SNPs were used for downstream processing. Applied criteria to retain SNP markers in analyses were: call frequencies ≥0.95, and minor allele frequency (MAF) ≥0.03. Imputation of missing genotypes was performed using fastPHASE (v1.2) to close gaps in the genotype matrix^53^. Marker genotype information was subsequently merged with the latest version of the S*us scrofa* build 10.2 available at http://www.animalgenome.org/repository/pig/ (2014-07-07). The map file contains markers mapping to all 18 porcine autosomes, both sex chromosomes and a contig of unmapped markers combined as chromosome ‘UWGS’, resulting in 51661 annotated SNPs used for downstream analyses.

## Association Analyses

### Genome-wide single-marker analyses

Mixed linear models implemented in JMP genomics 6 (SAS Institute, Cary, USA) were employed for multiple single-SNP-trait association analyses. The models included random effects of dam subline and sire subline to account for relatedness between individuals. To account for age related differences, linear models of SFT and LMP included growth rate as covariate. Regarding the setting of significance thresholds, the simpleM R script was used to estimate the number of independent tests^54^. Based on 23496 independent test (with the principal components accounting for 99.5% of the variance), thresholds were set to *p* = 4.3e-05 (1/23496) for suggestive significance and *p* = 2.1e-06 (0.05/23496) for genome-wide significance. Results were depicted as Manhattan plot using the qqman R package^55^.

### Genome-wide multi-marker analyses

All multi-marker analyses were performed using a Bayesian approach implemented in the web-based GenSel software (version 4.73 R)^56^. Parameters were set to a chain length of 51000 iterations including 1000 cycles as burn-in phase, and an output was created at 50 iteration intervals. The π-value, representing the number of SNPs considered as having no effects, was set to 0.995 as previously described for pigs^57^. Thus, approximately 260 SNPs were reported in a single iteration of the Markov chain Monte Carlo (MCMC) chain. Variance components were estimated using initial Bayes C analyses implemented in GenSel. Taking the estimated residual and genetic variance into account, the multi-marker approach was applied to the dataset employing a Bayes B algorithm. Values of growth rates were included as covariate in the analyses of SFT and LMP. Subsequently, the individual marker results were combined to estimate the contribution of non-overlapping 1-Mb windows to the genetic variance of the three analysed traits. In total, 2577 1-Mb windows were considered (excluding linkage group ‘UWGS’) resulting in a theoretical proportion to the genetic variance of a single window of about 0.04% (100%/2577). Windows that explain more than 0.5% of the genetic variance of a trait were considered in downstream investigations.

### Candidate gene selection and validation

Based on the integration of both genome-wide approaches, positional candidate genes were obtained using the porcine genome resource (Ensemble pig genome release 84, http://www.ensembl.org/Sus_scrofa). Moreover, corresponding genomic regions were screened for functional candidate genes using the information of the GeneCards database (http://www.genecards.org/). For promising positional and functional candidate genes, polymorphisms (tagging SNPs) were identified by sanger-sequencing of phenotypic-divergent individuals or by using the SNP database (dbSNP, http://www.ncbi.nlm.nih.gov/SNP/). Subsequently, DNA-based tools for genotyping polymorphisms of Solute Carrier Family 27 Member 6 (*SLC27A6*; rs342478551), Secreted Protein Acidic And Cysteine Rich (*SPARC*; rs319770026), and Bardet-Biedl Syndrome 7 (*BBS7*; rs320343985) were developed based on restriction fragment length polymorphism (RFLP) assays. Moreover, the *MC4R* polymorphism rs81219178, previously suggested to be causal for effects on fat deposition traits in pigs^15^, was analysed. In brief, RFLP assays were carried out in a standard PCR mix with SupraTherm Taq Polymerase (Genecraft, Lüdinghausen, Germany) according to manufactor’s specifications. Used primer pairs and restriction enzymes (all New England Biolabs, Frankfurt, Germany) are shown in Supplementary Table 1. Restriction fragments were separated on 2% agarose gel and analysed. Association analyses were performed for a representative subset of the population (n = 356) using SAS (MIXED procedure; SAS Institute). Models included dam subline and sire subline as random effects. Additionally, for the analyses of SFT and LMP, age was considered as a covariate.

### Data availability

The data that support the findings of this study are available from the corresponding author upon reasonable request.

## Electronic supplementary material

Supplementary Information
