# Supplementary material for: Genetics of body fat mass and related traits in a pig population selected for leanness
Source: Sci Rep. 2017 Aug 22;7:9118. doi: 10.1038/s41598-017-08961-4 (PMC5567295; doi:10.1038/s41598-017-08961-4)
Supplement: Supplementary file 1 — Supplementary Information [file 41598_2017_8961_MOESM1_ESM.doc]

**Genetics of body fat mass and related traits in a pig population selected for leanness**

Henry Reyer, Patrick F. Varley, Eduard Murani, Siriluck Ponsuksili, and Klaus Wimmers

**Supplementary Information**

**Supplementary Table 1**

Overview of single nucleotide polymorphisms (SNP) located in the coding region of selected candidate genes and specifications for their genotyping.

| **Gene** | **SNP** | **Region** | **Enzyme** | **Forward primer** | **Reverse primer** |
| --- | --- | --- | --- | --- | --- |
| SLC27A6 | rs342478551 | Intron | *DraI* | AACAGGCACTGCTTTTGTTGA | TCTGCATACATTAAAACACAGTTGG |
| SPARC | rs319770026 | Exon | *AflII* | TTCTATGTGCAGTGTCCTTCCC | ACTTGTCAGAACAGGGATGTGG |
| BBS7 | rs320343985 | 3’UTR | *BamHI* | AAACATAAATGAAATGAGTCTGATGG | ATCCCCCAGTCTGTTCAAGC |
| MC4R | rs81219178 | Exon | *TaqI* | ATGCTGGCTCTCATGGCTTC | TGATGCCTTACAAGAAAATGCTG |

**Supplementary Table 2**

Single nucleotide polymorphisms (SNP) with genome-wide significant evidence for association with lean mass percentage (LMP), subcutaneous fat thickness (SFT), and days to 110kg (D110) obtained from single marker (mixed linear model) analyses.

| **Trait** | **SNP_ID** | **Chromosome** | **Position (bp)** | **Major_allele** | **P-value** |
| --- | --- | --- | --- | --- | --- |
| D110 | ALGA0006298 | 1 | 162161768 | A | 2.82E-07 |
| D110 | ASGA0004976 | 1 | 176492950 | T | 4.00E-08 |
| D110 | ISU10000047 | 2 | 5561049 | T | 1.19E-06 |
| D110 | ALGA0084616 | 15 | 32933373 | A | 1.44E-06 |
| D110 | H3GA0044088 | 15 | 34303162 | C | 5.89E-07 |
| D110 | ALGA0108466 | 21 | 123156630 | A | 9.02E-08 |
| LMP | ALGA0005584 | 1 | 124845510 | T | 6.78E-07 |
| LMP | ALGA0005610 | 1 | 125359680 | C | 1.42E-06 |
| LMP | MARC0103791 | 1 | 125424741 | G | 1.42E-06 |
| LMP | ALGA0006564 | 1 | 175678980 | A | 6.48E-07 |
| LMP | ALGA0006566 | 1 | 175858119 | G | 4.98E-07 |
| LMP | ALGA0006599 | 1 | 177006345 | G | 1.72E-06 |
| LMP | ASGA0004992 | 1 | 177744561 | A | 2.56E-08 |
| LMP | ALGA0006623 | 1 | 178024855 | T | 1.32E-09 |
| LMP | INRA0004984 | 1 | 179188746 | T | 3.05E-07 |
| LMP | ALGA0103973 | 1 | 180494662 | G | 1.17E-06 |
| LMP | ASGA0004621 | 1 | 231022898 | C | 1.45E-06 |
| LMP | MARC0014974 | 5 | 103063418 | C | 1.94E-06 |
| LMP | MARC0019460 | 5 | 103084747 | A | 1.94E-06 |
| LMP | INRA0030146 | 8 | 100645138 | G | 1.41E-06 |
| LMP | CASI0009346 | 8 | 100889708 | A | 1.68E-06 |
| LMP | ASGA0039385 | 8 | 100921095 | T | 1.41E-06 |
| LMP | MARC0045606 | 8 | 102031078 | A | 1.34E-06 |
| LMP | ASGA0039389 | 8 | 102058185 | G | 6.38E-07 |
| LMP | ALGA0048723 | 8 | 102070107 | A | 6.38E-07 |
| LMP | M1GA0012006 | 8 | 102135908 | T | 1.34E-06 |
| LMP | ALGA0113149 | 13 | 25073183 | G | 1.96E-06 |
| LMP | ALGA0068831 | 13 | 25158584 | C | 2.03E-06 |
| LMP | ALGA0068836 | 13 | 25244249 | A | 2.03E-06 |
| LMP | ASGA0056657 | 13 | 25269531 | C | 2.03E-06 |
| LMP | ALGA0068846 | 13 | 25283128 | A | 2.03E-06 |
| LMP | ALGA0068855 | 13 | 25297815 | A | 2.03E-06 |
| SFT | MARC0063133 | 2 | 28893680 | G | 1.78E-06 |
| SFT | MARC0036560 | 5 | 68326348 | A | 2.09E-06 |
| SFT | ALGA0113503 | 8 | 94427398 | G | 8.47E-08 |
| SFT | ALGA0048677 | 8 | 98667343 | G | 9.19E-08 |
| SFT | INRA0030121 | 8 | 98942373 | A | 1.91E-07 |
| SFT | ASGA0039365 | 8 | 99027479 | G | 1.72E-07 |
| SFT | H3GA0025183 | 8 | 99050826 | G | 5.93E-07 |
| SFT | MARC0109916 | 8 | 99661929 | C | 1.06E-06 |
| SFT | H3GA0025190 | 8 | 99747955 | T | 3.60E-07 |
| SFT | ALGA0048710 | 8 | 100532519 | C | 4.60E-09 |
| SFT | ALGA0048717 | 8 | 100629167 | A | 1.45E-06 |
| SFT | INRA0030146 | 8 | 100645138 | G | 4.66E-10 |
| SFT | MARC0035880 | 8 | 100725819 | T | 3.20E-08 |
| SFT | H3GA0025193 | 8 | 100792448 | T | 2.63E-08 |
| SFT | CASI0009346 | 8 | 100889708 | A | 4.54E-10 |
| SFT | ASGA0039385 | 8 | 100921095 | T | 4.66E-10 |
| SFT | ASGA0100149 | 8 | 101266733 | G | 1.47E-07 |
| SFT | ASGA0039391 | 8 | 101976068 | G | 1.32E-07 |
| SFT | MARC0045606 | 8 | 102031078 | A | 3.40E-08 |
| SFT | ASGA0039389 | 8 | 102058185 | G | 2.04E-09 |
| SFT | ALGA0048723 | 8 | 102070107 | A | 2.04E-09 |
| SFT | M1GA0012006 | 8 | 102135908 | T | 3.40E-08 |
| SFT | DRGA0008716 | 8 | 102205112 | A | 1.96E-06 |
| SFT | MARC0056897 | 8 | 107706377 | A | 1.22E-08 |
| SFT | ALGA0119219 | 8 | 107724906 | G | 3.28E-09 |
| SFT | MARC0055736 | 8 | 107871574 | T | 7.74E-09 |
| SFT | ALGA0111705 | 8 | 107928428 | A | 7.74E-09 |
| SFT | ASGA0039446 | 8 | 108380457 | C | 1.30E-07 |
| SFT | ASGA0039463 | 8 | 109331726 | A | 1.96E-09 |
| SFT | MARC0071612 | 8 | 109378869 | T | 9.41E-08 |
| SFT | ALGA0122904 | 8 | 109553065 | C | 5.20E-11 |
| SFT | ALGA0048908 | 8 | 109609357 | T | 2.05E-08 |
| SFT | H3GA0025268 | 8 | 112440225 | G | 1.61E-06 |
| SFT | ASGA0039589 | 8 | 116982940 | G | 5.44E-08 |
| SFT | ALGA0049119 | 8 | 117393889 | A | 6.03E-08 |
| SFT | ASGA0039614 | 8 | 117902625 | T | 5.62E-07 |
| SFT | ALGA0049173 | 8 | 117981832 | G | 7.95E-07 |
| SFT | ALGA0083738 | 15 | 259597 | A | 4.42E-07 |
